# Optimization of Ultra-High Performance Concrete Based on Response Surface Methodology and NSGA-II

**DOI:** 10.3390/ma17194885

**Published:** 2024-10-04

**Authors:** Zhenxing Wang, Jiaming Wu, Lei Su, Zhaolin Gao, Chenglin Yin, Zhengmao Ye

**Affiliations:** 1 School of Materials Science and Engineering, University of Jinan, Jinan 250022, China; jdclwzx@stu.ujn.edu.cn (Z.W.); 19861821793@163.com (Z.G.); yin_chlin@163.com (C.Y.); 2 Tiezheng Testing Technology Co., Ltd., Jinan 255001, China; leisu_2002@163.com

**Keywords:** the response surface methodology, UHPC, mechanical performance, workability, NSGA-II

## Abstract

This study systematically investigated three influential factors—water-to-binder ratio, cement/sand ratio, and steel fiber content—that significantly impact the performance of ultra-high-performance concrete (UHPC). Utilizing the Response Surface Methodology (RSM) with a Central Composite Design (CCD), 20 carefully designed mix proportions underwent comprehensive experimental testing. Through rigorous statistical analysis, models were established to elucidate the complex relationships between the specified factors and the overall properties of UHPC. Variance analysis reveals significant effects of the three factors on UHPC performance, with workability and compressive strength increasing with higher cement/sand ratios while flexural strength decreases. Moreover, increased water-to-binder ratios exhibit substantial negative impacts on both 28-day compressive and flexural strengths. Despite adversely affecting workability, higher steel fiber dosages contribute positively to mechanical performance. Furthermore, Monte Carlo sampling and the multi-objective non-dominated sorting genetic algorithm-II (NSGA-II) were employed to validate the reliability of the statistical model and to conduct multi-objective optimization. The final UHPC mix design obtained consists of a cement/sand ratio of 1.12, a water/binder ratio of 0.16, and a steel fiber content of 2.94%. Experimental results yielded a slump flow of 802 mm, compressive strength of 122.7 MPa, and flexural strength of 24.3 MPa.

## 1. Introduction

Since its inception, Ultra-High-Performance Concrete (UHPC) [1] has witnessed a growing significance in the realm of construction engineering, evolving over time. Notably, the realm of construction has articulated a demand for the high-performance characteristics of building materials to effectively address challenges posed by adverse conditions [2,3]. The extensive utilization of UHPC in practical engineering is attributable to its remarkable mechanical properties and exemplary durability, including its capacity to withstand the deleterious effects of substance erosion [4,5].

In comparison to ordinary concrete, UHPC utilizes a high cement content (800–1000 kg/m^3^) [6], with the dosage of silica fume accounting for 25–35% of the cement content [7]. Fine aggregates ranging from 120 to 800 μm are chosen, and UHPC typically employs a low water-to-binder ratio. The use of efficient superplasticizers ensures workability [8], and 13 mm long, 0.2 mm diameter steel fibers are added. The mix design is conducted based on the closely packed theory to achieve the densest state and, consequently, an ultra-low porosity, leading to enhanced performance [9,10,11,12,13,14]. The water-to-binder ratio, cement/sand ratio, and steel fiber content are critical factors exerting significant influence on the mechanical properties of UHPC [15,16].

The Response Surface Method (RSM) is an analytical approach that combines mathematical and statistical methods for optimizing multi-factor parameters. In contrast to single-factor and orthogonal experimental methods, RSM considers interactions among multiple factors to predict the optimal response values. The optimal results obtained from RSM are not limited to a specific set of experiments but rather represent the optimal points on the entire three-dimensional surface. RSM analyzes the interactions between major influencing factors by fitting models and establishes specific functional relationships between influencing factors and response values, thereby enabling discussions on the extent of the impact of multiple factors on response values. Consequently, it finds extensive application in the fields of multi-parameter optimization and material design [17,18,19,20]. In recent years, with the rapid and continuous development of the concrete industry, RSM has found widespread use in the optimization of concrete configurations for multiple performance requirements. It achieves this by adjusting certain nonlinear factors to define an optimal domain [21]. Many scholars have utilized RSM to study the optimization of UHPC.

Ferdosian et al. [22], employing the Response Surface Methodology (RSM), investigated the impact of sand, silica fume, and ultrafine fly ash on the primary properties of UHPC. They utilized multi-objective optimization to obtain the optimal mix proportions for enhanced performance. Wille et al. [23] studied the influence of parameters such as cement, silica fume, and water-to-binder ratio on UHPC performance, ultimately determining the optimal range for these parameters. Mohammed et al. [24] explored the effects of different water-to-binder ratios on UHPC performance, revealing that lower ratios resulted in higher mechanical performance. Steel fibers exert a significant influence on the toughness and flexural performance of UHPC, with current research indicating that the highest acceptable fiber content is 6% [16]. The type of steel fiber also imparts varying effects on UHPC; for a given content, shaped steel fibers exhibit a greater impact on tensile and flexural strength than straight steel fibers [25]. Additionally, different lengths of steel fibers contribute differently to UHPC performance [26]. Mu et al. [27] applied a magnetic field to orient the steel fibers in UHPC, enhancing its mechanical properties. Wang et al. [28] adjusted the water-to-binder ratio or the dosage of superplasticizers to achieve different rheological properties, studying the influence of rheological properties on fiber distribution and yield stress.

Due to the complexity of UHPC compositions, minor perturbations can result in significant performance deviations. Currently, multiple experiments are typically conducted to reduce experimental errors, leading to a substantial increase in the number of experiments required. NSGA-II is a commonly used multi-objective optimization algorithm. It simulates the process of natural evolution to find the optimal solution set while maintaining a balance between different objectives. In recent years, the powerful optimization capability of NSGA-II has led to its widespread application in the research of cement-based materials [29,30].

Many researchers have extensively studied the influencing factors of UHPC using Response Surface Methodology (RSM). However, there is a lack of systematic research utilizing both RSM and NSGA-II algorithms for optimizing UHPC mix proportions. This study primarily focuses on exploring the collective effects of the water-to-binder ratio, cement/sand ratio, and steel fiber content. The research objectives involve optimizing these factors using the Central Composite Design (CCD) method within the framework of Response Surface Methodology, with workability, compressive strength, and flexural strength selected as response variables. Regression analysis, residual analysis, and model fitting tests were employed for a thorough analysis of each factor, combining the NSGA-II method for multi-objective optimization; the ultimate aim was to derive a well-optimized mix proportion for UHPC.

## 2. Materials and Methods

### 2.1. Materials

**Figure 1:**
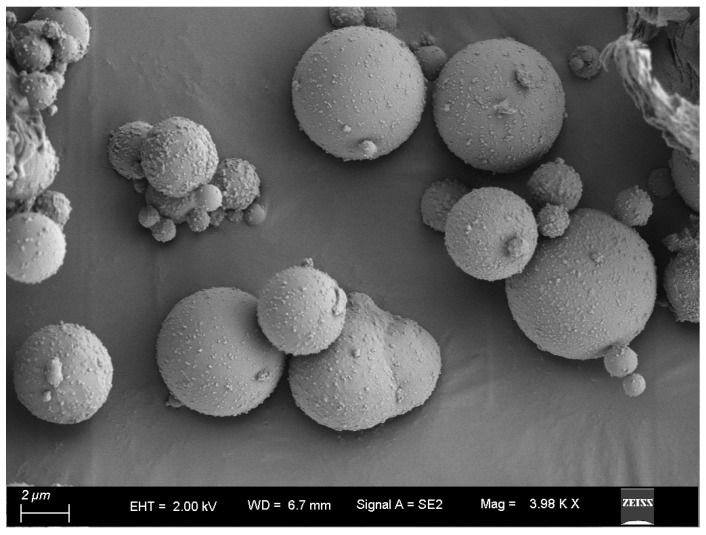
Micrographs of microspheres.

**Figure 2:**
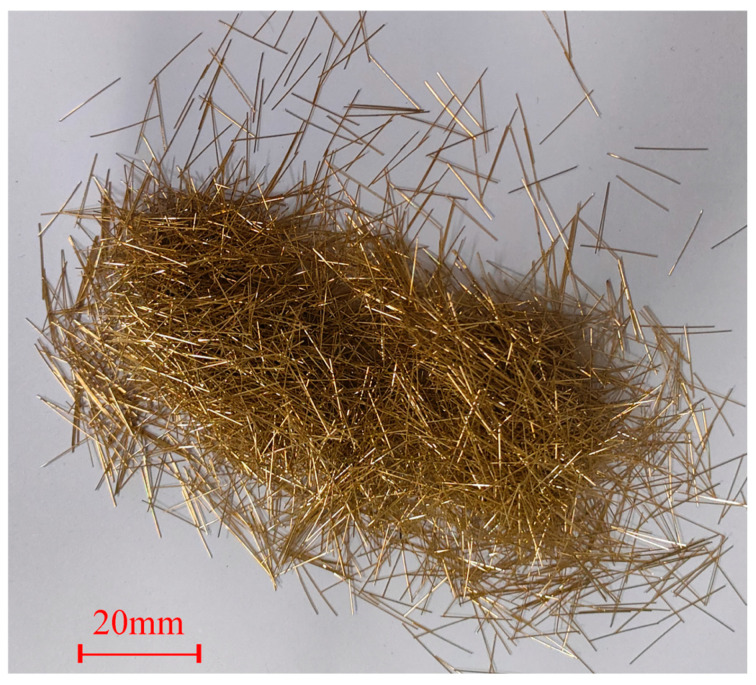
Steel Fibers.

In this study, the raw materials used include PO 52.5 Portland cement, microspheres (such as in Figure 1), silica fume (SF), an expanding agent, steel fiber, quartz sand, and a polycarboxylate acid water reducer. The chosen fibers are straight steel fibers with a diameter of 0.2 mm and a length of 1.3 mm (such as in Figure 2). Two grades of quartz sand, namely 20–40 mesh and 40–80 mesh, were used. Microspheres are spherical particles with a continuous and uniform particle size distribution, selected from fly ash, with a particle size smaller than 30 microns, the majority of which ranges from 0.1 μm to 5 μm. The incorporation of microspheres during concrete preparation results in excellent optimization of the concrete gradation at the microscopic level, leading to increased density and higher strength. Table 1 provides the chemical compositions of cement, microspheres, and silica fume.

### 2.2. Specimens Preparation

**Figure 3:**
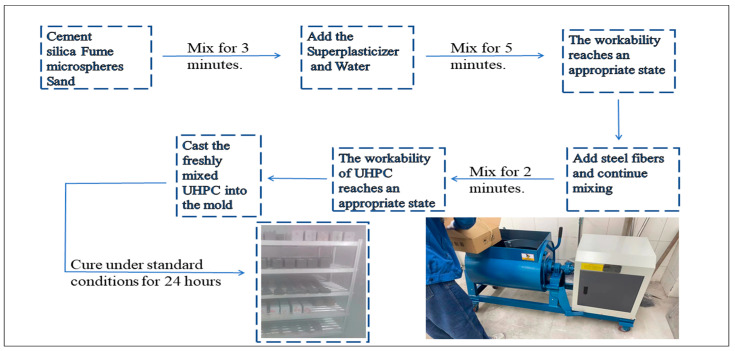
Preparation of the UHPC mixtures.

The preparation of test specimens involves the following steps: Initially, mix the cement and aggregates, such as quartz sand, for 3 min. Subsequently, add the high-efficiency water-reducing agent and water and stir for 5 min. Once the mixture reaches an appropriate consistency, uniformly disperse the steel fibers by adding them and stirring for an additional 2 min (such as in Figure 3). After the mixing process is completed, conduct slump flow tests and mold specimens according to the Chinese standard T/CECS 864 [31].

### 2.3. Experimental Design and RSM

The RSM statistical method can achieve optimized results for target performance under given influencing conditions and can significantly minimize the number of experiments required. An experimental design was conducted based on the requirements of Response Surface Methodology (RSM) and the key performance factors of UHPC, following the Central Composite Design (CCD) approach.

As shown in Table 2 in this study, the variables selected are cement/sand ratio (coded as A), water-to-binder ratio (coded as B), and steel fiber content (coded as C). Table 3 shows the specific mix design. The response values are slump flow, compressive strength, and flexural strength, as indicated in Table 4.

### 2.4. Experimental Methods

#### 2.4.1. Workability

**Figure 4:**
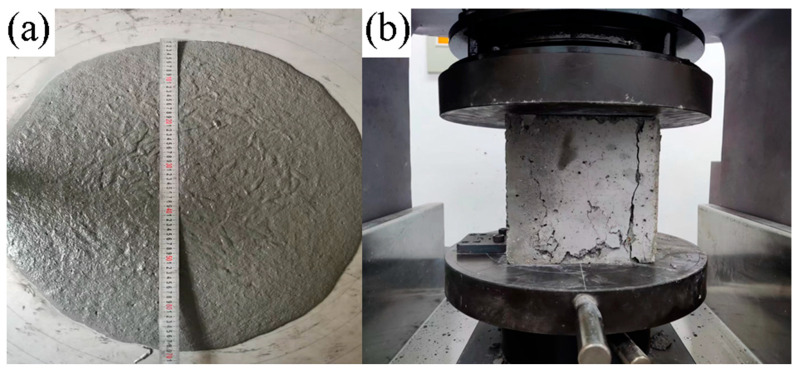
The working (**a**) and mechanical (**b**) performance of UHPC samples.

Following the Chinese Standard T/CECS 864 for the test method of ultra-high performance concrete, the workability performance of freshly mixed UHPC is evaluated using the slump flow test. A slump cone is filled in one continuous operation, and excess material is struck off level with the top of the cone. The cone is then smoothly and vertically lifted, and the diameter of the spread of UHPC is measured in two perpendicular directions using a steel ruler along the edge of the cone. For example, Figure 4a illustrates the testing process.

#### 2.4.2. Compressive Strength

The compressive strength test follows the Chinese T/CECS 864 Standard for the test method of ultra-high performance concrete, which involves casting the specimens into molds with dimensions of 100 × 100 × 100 mm^3^. Immediately after cast, the specimens are covered with plastic film and cured for 24 h at 20 ± 2 °C. After demolding, the specimens are further cured at a temperature of 20 ± 2 °C and a relative humidity exceeding 95%. The compressive strength test for UHPC cubic specimens is conducted at a curing age of 28 days, with six specimens per batch.

#### 2.4.3. Flexural Strength

Following the Chinese T/CECS 864 Standard for test method of ultra-high performance concrete, form 100 × 100 × 400 mm^3^ prismatic specimens. After demolding, place the specimens under cured conditions at a temperature of 20 ± 2 °C and a relative humidity exceeding 95%. When the cured period reaches 28 days, conduct the flexural strength test with three specimens per batch.

## 3. Results and Analysis

In this study, the CCD model consists of three factors with three levels each and three response values: slump flow, compressive strength at 28 days, and flexural strength at 28 days. The summarized test results are presented in Table 4. The compressive strength data represent the arithmetic mean of six specimen measurements, meeting the standard requirement that the individual test values deviate no more than 10% from the mean. The flexural strength data are the arithmetic mean of three measurements, with deviations not exceeding the 15% standard requirement.

In Table 5, the results of the regression model and the variance analysis are presented. Parameters associated with significance, such as the F-value, *p*-value, R-squared (R^2^), and Adjusted R-squared (Adj-R^2^), are outlined in the ANOVA table (factors with excessively high *p*-values have been removed in Table 5). A *p*-value less than 0.05 indicates that the model term is significant; conversely, a *p*-value greater than 0.1 indicates that the model term is not significant, with all model *p*-values being <0.0001. Utilizing regression analysis, the final model is derived.

The evaluation criteria for UHPC performance prioritize compressive strength and flexural strength as the primary indicators, while workability serves as a secondary indicator. The primary objective is to achieve high compressive and flexural strengths, followed by the goal of obtaining satisfactory workability.

### 3.1. Analysis of Working Performance

**Figure 5:**
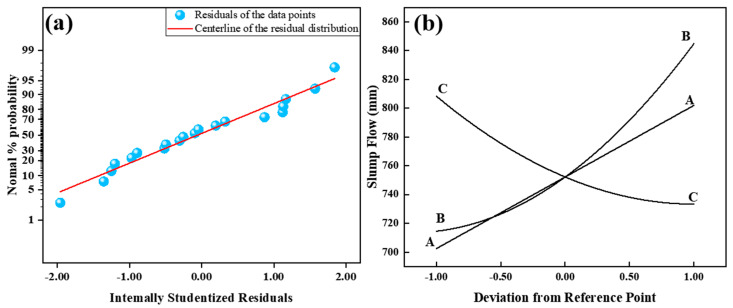
Normal plot of residuals value (**a**) and perturbation curve of Slump Flow (**b**).

In Table 4, the actual and predicted values of UHPC workability analyzed through RSM are presented. The non-significant data for Lack of Fit (F = 4.71) suggests that the model is suitable for predictive analysis (R^2^ = 0.9333; Adj. R^2^ = 0.9094). Figure 5a illustrates the normal distribution plot of standardized residuals for slump flow. The residuals closely align with the straight line, indicating a satisfactory fit of the model.

According to the ANOVA results in Table 5, the relationship between workability and the three factors has been determined. The ANOVA model used is as follows:

Slump flow of UHPC mm = 752.26 + 49.76 × A + 65.17 × B − 37.51 × C + 27.48 × B^2^ + 18.64 × C^2^.

where A represents the cement/sand ratio, B represents the water-to-binder ratio, and C represents the steel fiber content.

**Figure 6:**
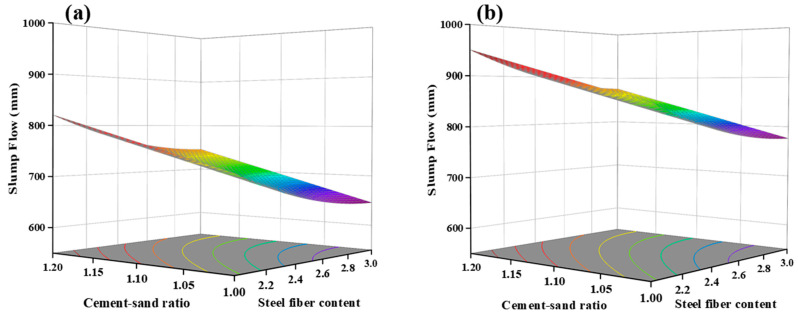
Slump flow response surface with water-to-binder ratio: (**a**) Low water-to-binder ratio (0.14), (**b**) High water-to-binder ratio (0.18).

As depicted in Figure 5b, the component effects curve illustrates the variation trend of model-predicted response values with changes in variables. The impact of factor A on slump flow is linear, while the effects of factors B and C are nonlinear. With an increment in the cement/sand ratio and water-to-binder ratio, the slump flow of freshly mixed UHPC increases. The rise in the water-to-binder ratio leads to increased moisture surrounding the cement particles, resulting in an augmented water film thickness and reduced inter-particle friction, consequently enhancing the slump flow [32]. From Figure 6, it can be inferred that, with a steel fiber content of 3.0% and a cement/sand ratio of 1.2, the maximum increase in slump flow is 160 mm with an increasing water-to-binder ratio (Such as samples 1 and 10).

**Figure 7:**
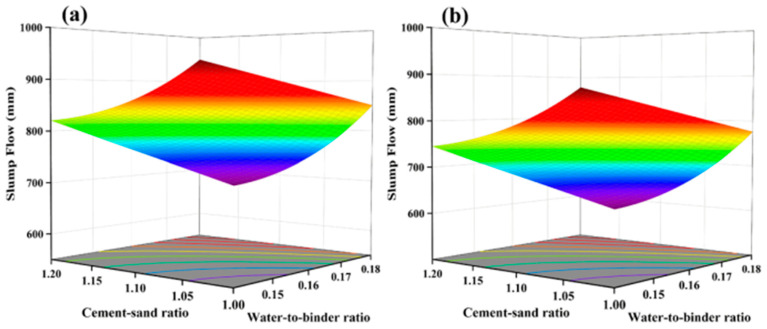
Slump flow response surface with steel fiber content: (**a**) Low steel fiber content (2%), (**b**) High steel fiber content (3%).

Due to the mechanical fragmentation of quartz sand, its particles exhibit numerous sharp edges and irregular shapes, impeding the flow of UHPC. With an increase in the cement/sand ratio, the volume of quartz sand per unit of UHPC decreases, mitigating this hindrance and resulting in an augmented slump flow. From Figure 5b and Figure 7, the impact of factor C, steel fiber content, on slump flow is detrimental. As the fiber content increases, slump flow decreases, with a maximum reduction of up to 120 mm. This phenomenon arises from the crisscross arrangement of steel fibers within the UHPC, forming a distinct network that impedes the flow. Greater steel fiber content leads to a denser network with smaller mesh sizes, thereby intensifying the hindering effect.

### 3.2. Analysis of Compressive Strength Performance

**Figure 8:**
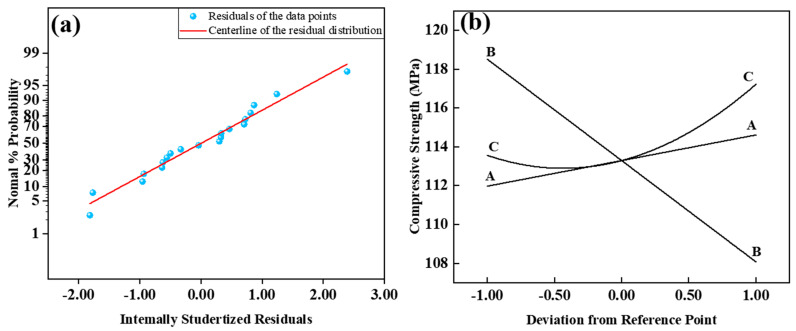
Normal plot of residual value (**a**) and perturbation curve of 28-day compressive strength (**b**).

In Table 4, the actual and predicted values of UHPC’s 28-day compressive strength are presented through RSM analysis. The non-significant data for Lack of Fit (F = 1.92) suggests the applicability of the model for predictive analysis (R^2^ = 0.8806; Adj.R^2^ = 0.8380). Figure 8a depicts the normal distribution plot of standardized residuals for the 28-day compressive strength. The proximity of residual values to the straight line indicates favorable results. Based on the ANOVA results in Table 5, the relationship between workability and the three factors is established. The ANOVA model employed is as follows:

The 28-day compressive strength of UHPC MPa = 113.29 + 1.32 × A − 5.22 × B + 1.83 × C − 1.95 × AC + 2.10 × C^2^.

The *p*-value of factor A in the model is 0.0510, which is less than the predetermined significance level, but considering the small difference between the *p*-value and 0.05, it still has certain statistical significance, so it is not ignored.

As shown in Figure 8b, the component effect curves illustrate the trend of the predicted response values with varying variables. The impact of factors A and B on compressive strength is linear, while the influence of C on compressive strength is nonlinear. With an increase in the cement/sand ratio, the 28-day compressive strength exhibits a linear improvement. This result may be related to the Interfacial Transition Zone (ITZ) around the aggregate, which is considered a weak area in UHPC. An increase in the cement/sand ratio leads to a decrease in the volume fraction of aggregates. As the amount of aggregates in UHPC decreases, the ITZ formed in the cured UHPC reduces. Although the reduction in aggregates weakens the compressive strength of UHPC, the decrease in the ITZ enhances the compressive strength. These two effects counteract and combine, increasing compressive strength [33].

**Figure 9:**
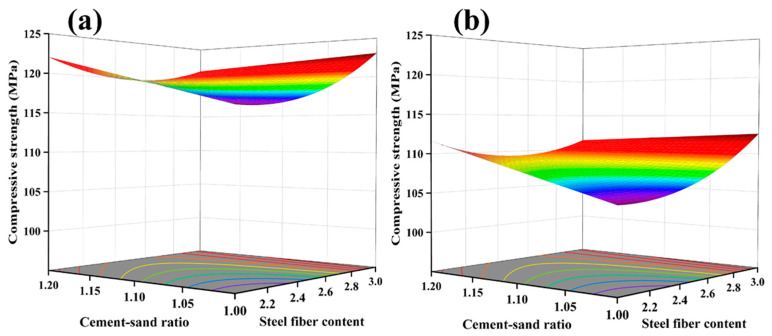
Response Surface of 28-day Compressive Strength as a Function of water-to-binder Ratio: (**a**) Low water-to-binder Ratio (0.14), (**b**) High water-to-binder Ratio (0.18).

With an increase in the water-to-binder ratio, the compressive strength of all UHPC specimens shows a significant decrease trend. This is consistent with the research results of Appa Rao [34], Mohamad, et al. [35], and Fernandes et al. [36]. An increase in the water-to-binder ratio leads to a thicker water film on the particle surfaces, resulting in more pores in the cured UHPC, increasing the porosity, and weakening the ITZ. As shown in Figure 9, with a constant steel fiber content and cement/sand ratio, an increase in the water-to-binder ratio causes the 28-day compressive strength to decrease from 124.6 MPa to 113.9 MPa, a reduction of 10.7 MPa.

**Figure 10:**
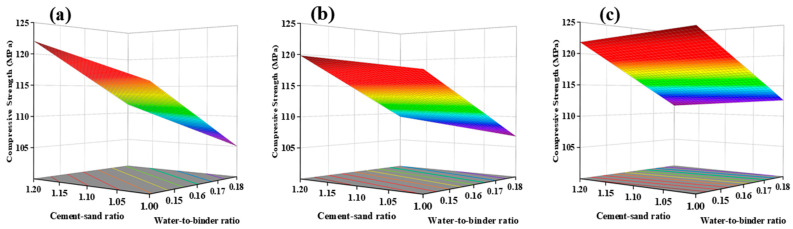
Response Surface of 28-day Compressive Strength as a Function of Steel Fiber Content: (**a**) Low Steel Fiber Content (2%), (**b**) Medium Steel Fiber Content (2.5%), (**c**) High Steel Fiber Content (3%).

The influence of steel fibers on the 28-day compressive strength shows a decreasing-then-increasing trend with an increase in the content. The lowest point occurs at a content of 2.50%, as shown in Figure 10. This result may be attributed to the hindrance effect of steel fibers on the flow of UHPC, leading to a decrease in compactness, thereby reducing strength when the fiber content increases from 2.0% to 2.5% [37]. When the content increases from 2.5% to 3.0%, the increase in strength due to steel fibers outweighs the impact of voids, increasing compressive strength.

### 3.3. The Flexural Strength Performance Analysis

**Figure 11:**
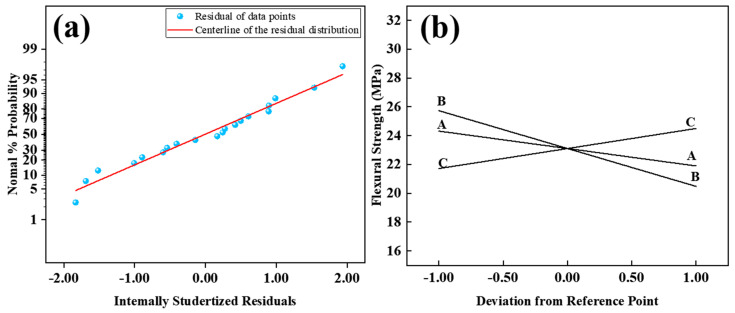
Normal plot of residual value (**a**) and perturbation curve (**b**) of Flexural Strength.

In Table 4, the actual values and predicted values of UHPC flexural strength through Response Surface Methodology (RSM) analysis are presented. The non-significant Lack of Fit data (F = 2.08) indicates the suitability of the model for predictive analysis (R^2^ = 0.7298; Adj.R^2^ = 0.6792). Figure 11a illustrates the normal distribution of standardized residuals for flexural strength. The residuals closely align with the straight line, indicating satisfactory results. Based on the ANOVA results in Table 5, the relationship between flexural strength and the three factors is established. A linear relationship is observed between flexural strength and the three factors, and the ANOVA model used is as follows:

Flexural Strength of UHPC MPa = 23.11 − 1.21 × A − 2.63 × B + 1.39 × C.

As shown in Figure 11b, the component effect curves illustrate the trend of predicted response values with changes in components. The influence of the three factors on flexural strength is linear. Flexural strength exhibits a negative correlation with factors A and B, where the cement/sand ratio and water-to-binder ratio gradually increase from low levels (coded as −1) to high levels (coded as +1), resulting in a decrease in flexural strength. An increase in the water-to-binder ratio leads to more internal pore structures and a decrease in the strength of the ITZ. The increase in the cement/sand ratio leads to a decrease in flexural strength, which can be explained by the crack bridging mechanism [38,39]. The interlocking of sand particles inside the specimen significantly consumes the energy of the crack surface. When the crack encounters materials similar to sand during the expansion process, it cannot pass directly through the sand particles but has to transfer energy along the transition zone on the surface of the sand particles. This increases the energy transfer path, requiring the consumption of more energy. Therefore, an increase in the cement/sand ratio leads to a decrease in sand content, resulting in a reduction in flexural strength.

**Figure 12:**
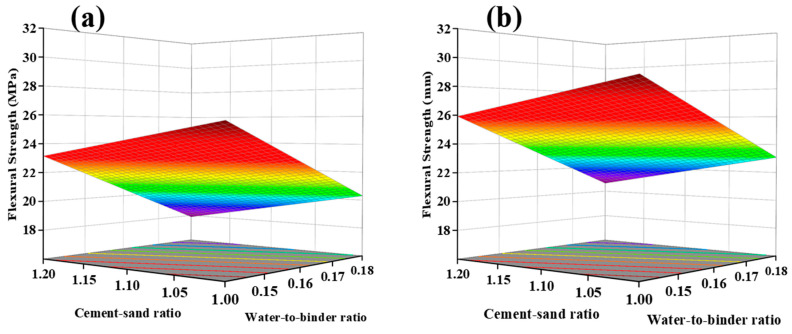
Response Surface of 28-day Flexural Strength with Steel Fiber Content: (**a**) Low Steel Fiber Content, (**b**) High Steel Fiber Content.

The impact of factor C, in contrast, exhibits an opposing trend to A and B, showing a positive correlation between flexural strength and steel fiber content. The increasing content of steel fiber results in smaller inter-fiber spacing and denser microstructures near the fibers. Consequently, a better bond strength forms between the fibers and the matrix [40]. When cracks occur, more steel fibers intersect each crack, increasing the bonded area between the matrix and the fibers. This impedes the extension and propagation of cracks, leading to the formation of more microcracks and delaying the growth of macroscopic cracks [25,41]. As a result, a higher initial cracking strength is achieved, leading to higher flexural strength of UHPC. Consequently, higher flexural strength in UHPC can be achieved by introducing a greater concentration of steel fibers. This observation is consistent with the pattern illustrated in Figure 12, where flexural strength rises with an augmentation in steel fiber content while keeping other variables constant. For instance, in comparison between UHPC samples with 2% and 3% steel fiber content (cement/sand ratio of 1.0, water-to-binder ratio of 0.14), the flexural strength increases from 24.02 MPa to 27.51 MPa, representing a notable increase of 3.49 MPa.

### 3.4. Optimization Validation

#### 3.4.1. Constraints in Multi-Objective Optimization

The main purpose of multi-objective optimization is to minimize the mean square deviation while satisfying constraints and maximizing the reliability of the optimization results. Optimization methods effectively transform deterministic optimization into reliability-based optimization to find the optimal design space between the target performance and reliability probability of UHPC. Therefore, the design variables, optimization objectives, and constraints for the multi-objective optimization design based on robustness for UHPC are determined as follows:

Design Variables: Water-to-binder ratio, cement/sand ratio, and steel fiber content.

Optimization Objectives: (1) Slump flow, (2) Maximum 28-day compressive strength, (3) Maximum 28-day flexural strength.

Constraint Conditions: Deterministic constraints for slump flow range from 750 mm to 850 mm, for 28-day compressive strength range from 115 MPa to 125 MPa, and for 28-day flexural strength range from 21 MPa to 31 MPa. Therefore, based on the design variables, optimization objectives, and constraint ranges, the multi-objective optimization model is determined as follows:

$$\mathrm{Independent}\;\mathrm{variables}\;\left\{\begin{array}{c} 1.0\leq A\leq 1.2\\ 0.14\leq B\leq 0.18\\ 2\leq C\leq 3 \end{array}\right.$$

$$\mathrm{Constraint}\;\mathrm{range}\left\{\begin{array}{c} 750\leq \mathrm{Slump}\;\mathrm{flow}\leq 850,SF\in 0.01\times N\\ 115\leq \mathrm{Compressive}\;\mathrm{strength}\leq 125,CS\in 0.01\times N\\ 21\leq \mathrm{Flexural}\;\mathrm{strength}\leq 31,FS\in 0.01\times N \end{array}\right.$$

#### 3.4.2. Reliability Analysis

**Figure 13:**
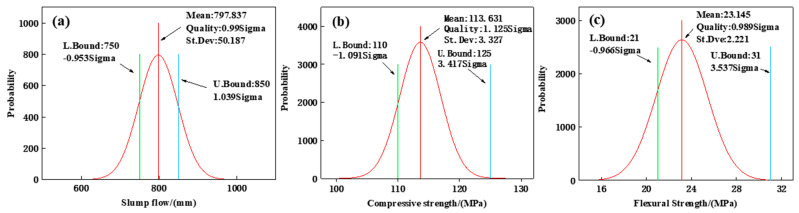
The Monte Carlo sampling analysis results: (**a**) results of Slum flow, (**b**) results of Compressive strength, (**c**) results of Flexural strength.

The reliability analysis was conducted using the Monte Carlo sampling method. Random factors such as noise are selected, and 10,000 random samples are drawn to determine their distribution characteristics, including mean, standard deviation, and Sigma levels. As shown in Figure 13, the Sigma levels for slump flow, 28-day compressive strength, and 28-day flexural strength were determined to be 0.99, 1.125, and 0.99, respectively, with reliabilities of 67.93%, 86.94%, and 83.27%, all of which are below 90%. This indicates that the reliability of results within the deterministic constraint range based on the response surface model is relatively low.

#### 3.4.3. Pareto Frontier of Response Variables and Reliability Analysis

**Figure 14:**
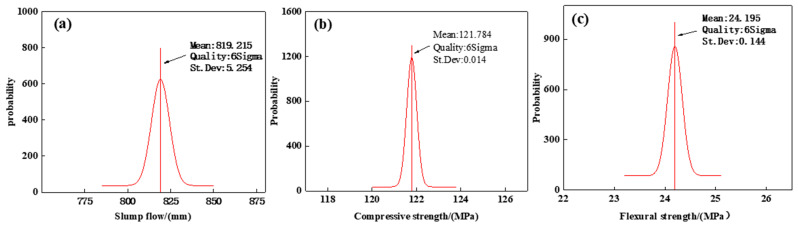
Reliability requirements of NSGA-II: (**a**) results of slum flow, (**b**) results of compressive strength, (**c**) results of flexural strength.

Based on the mathematical models in Section 3.1, Section 3.2 and Section 3.3, this study employed NSGA-II for multi-objective optimization. During optimization, the initial population size of NSGA-II was set to 200, with 200 iterations. The crossover distribution index was set to 0.8, mutation percentage to 0.7, and mutation probability to 0.4. NSGA-II operates by searching for the optimal Pareto solutions in the solution space and then performs Pareto front ranking to select the best solutions. Concurrently, to validate the reliability of the NSGA-II multi-objective optimization, reliability analysis was conducted on the sample data to obtain the mean, variance, and 6-sigma level, as shown in Figure 14.

From Figure 14, it can be observed that the sigma level values of the selected sample points for UHPC multi-objective optimization are all not less than 6. This indicates that the reliability of multi-objective optimization is not less than 99.99%, satisfying the reliability requirements of 6-sigma robust multi-objective optimization principles.

#### 3.4.4. Multi-Objective Optimization Result Verification

As shown in Table 6, based on the NSGA-II multi-objective optimization results, the ideal point was selected for validation experiments, with a cement/sand ratio of 1.12, a water-to-binder ratio of 0.16, and a steel fiber content of 2.94. This scheme was subjected to three repeated validation experiments. From the validation experiment results, it can be observed that there is a slight deviation between the experimental results and the NSGA-II optimization results. Although there is some deviation in the fitted model, the validation experiment results partially demonstrate the rationality of the optimization method.

## 4. Conclusions

This study employed the response surface methodology to analyze the influence of water-to-binder ratio, cement/sand ratio, and steel fiber content on the workability, 28-day compressive strength, and 28-day flexural strength of UHPC. The study also conducted multi-objective optimization.

Polynomial models for workability, compressive strength, and flexural strength were established. The fitting effect of the models was found to be satisfactory through various correlation coefficients and predicted values. Simultaneously, NSGA-II was employed for multi-objective optimization, and experimental validation confirmed that the optimized results closely matched the validation results.

The water-to-binder ratio exhibited a negative impact on 28-day compressive strength and flexural strength, with a more significant effect as the water-to-binder ratio increased, leading to a gradual reduction in mechanical properties.

The cement/sand ratio demonstrated a good linear correlation with the three response variables. Specifically, workability and compressive strength increased with an increase in the cement/sand ratio, while flexural strength decreased.

The study revealed that using a high dosage of steel fibers, while negatively affecting workability, proved beneficial for mechanical properties, especially in achieving higher flexural strength. The addition of 3% steel fibers was considered the most suitable dosage in UHPC, providing higher mechanical performance while maintaining adequate workability.

## Figures and Tables

**Table 1:** Chemical composition of cementitious materials (%).

Code	CaO	SiO_2_	Al_2_O_3_	Fe_2_O_3_	SO_3_	K_2_O	Na_2_O	MgO	Loss
Cement	63.06	21.01	3.76	3.34	3.63	0.89	0.19	3.03	2.1
Silica fume	0.35	97.43	0.42	0.13	0.33	0.16	0.33	0.22	1.2
microspheres	7.9	45.28	39.76	2.9	0.47	0.91	0.42	0.18	1.8

**Table 2:** Range and Codes of Variables in the CCD Method.

Variables	Coded	Actual		
		−1	0	1
Cement/sand ratio	A	1.00	1.10	1.20
Water-to-binder ratio	B	0.14	0.16	0.18
Steel Fiber Content (%)	C	2.00	2.50	3.00

**Table 3:** Mix Design in CCD Method.

Sample	Actual			Cement	Silica Fume	Microspheres	Sand		Superplasticizer	Steel Fiber	Water
	C/S	W/B	SFC				20–40	40–80			
1	1.20	0.18	3.00	865	110	110	275	629	28	234	195.3
2	1.10	0.16	1.66	865	110	110	300	687	28	129.5	173.6
3	1.20	0.18	2.00	865	110	110	275	629	28	156	195.3
4	1.10	0.16	2.50	865	110	110	300	687	28	195	173.6
5	1.00	0.14	3.00	865	110	110	330	755	28	234	151.9
6	1.10	0.13	2.50	865	110	110	300	687	28	195	141.1
7	1.00	0.14	2.00	865	110	110	330	755	28	156	151.9
8	1.10	0.19	2.50	865	110	110	300	687	28	195	206.2
9	1.10	0.16	2.50	865	110	110	300	687	28	195	173.6
10	1.20	0.14	3.00	865	110	110	275	629	28	234	151.9
11	1.10	0.16	2.50	865	110	110	300	687	28	195	173.6
12	1.27	0.16	2.50	865	110	110	260	595	28	195	173.6
13	0.93	0.16	2.50	865	110	110	355	812	28	195	173.6
14	1.00	0.18	2.00	865	110	110	330	755	28	156	195.3
15	1.10	0.16	2.50	865	110	110	300	687	28	195	173.6
16	1.10	0.16	3.34	865	110	110	300	687	28	160.5	173.6
17	1.20	0.14	2.00	865	110	110	275	629	28	156	151.9
18	1.10	0.16	2.50	865	110	110	300	687	28	195	173.6
19	1.00	0.18	3.00	865	110	110	330	755	28	234	195.3
20	1.10	0.16	2.50	865	110	110	300	687	28	195	173.6

where C/S: cement/sand ratio, W/B: Water-to-binder ratio, SFC: Steel Fiber Content.

**Table 4:** Experimental Results and Predicted Values of UHPC.

Sample	Workability (mm)	28 d-Compressive Strength (MPa)	28 d-Flexural Strength (Mpa)
1	900	112.6	21.59
2	860	116.6	23.17
3	930	110.5	18.53
4	720	114.3	24.68
5	670	124.6	27.51
6	700	122	30.55
7	720	116.1	24.02
8	920	101.1	16.33
9	740	111.2	23.54
10	740	118.6	23.09
11	760	114	24.85
12	840	120.1	21.5
13	650	110	24.93
14	870	106.5	19.68
15	750	112.2	21.54
16	710	121.8	26.84
17	860	120.4	20.54
18	780	116	23.99
19	810	113.9	23.34
20	745	111.9	22.06

**Table 5:** Results of the Full Regression Model and Variance Analysis from Numerical Simulation.

ANOVA	Responses					
	Slump Flow	*p*-Value	28 d-Compressive Strength (MPa)	*p*-Value	28 d-Flexural Strength (Mpa)	*p*-Value
intercept	+752.26		+113.29		+23.11	
Linear terms						
A	+49.76	<0.0001	+1.32	0.0510	−1.21	0.0245
B	+65.17	<0.0001	−5.22	<0.0001	−2.63	<0.0001
C	−37.51	<0.0001	+1.83	0.0103	+1.39	0.0119
Quadratic terms						
A2						
B2	+27.48	0.001				
C2	+18.64	0.0141	+2.10	0.0034		
Interaction terms						
AB						
AC			−1.95	0.0298		
BC						
Other terms						
R^2^	0.9333		0.8806		0.7298	
Adj-R^2^	0.9094		0.8380		0.6792	
Pred-R^2^	0.8245		0.7531		0.5405	
F-Value	20.66		20.66		14.41	
Lack of Fit	1.92		1.92		2.08	
Model *p*-Value	<0.0001		<0.0001		<0.0001	

**Table 6:** The validation experiment results of NSGA-II.

Response Quantity	Slump Flow/mm	Relative Error	28 d-Compressive Strength/MPa	Relative Error	28 d-Flexural Strength/MPa	Relative Error
Predicted value	815	—	123.6	—	24.4	—
Test 1	800	−1.84%	122.3	−1.05%	25.2	3.28%
Test 2	795	−2.45%	124.1	0.4%	24.1	−1.23%
Test 3	810	−0.6%	121.7	−1.54%	23.6	−4.51%
average	802	−1.6%	122.7	−0.73%	24.3	−0.41%

## Data Availability

The original contributions presented in the study are included in the article, further inquiries can be directed to the corresponding authors.
